# Association between the atherogenic index of plasma and high risk of obstructive sleep apnea in aged person over 60: A cross-sectional analysis based on NHANES 2017 to 2020

**DOI:** 10.1097/MD.0000000000044665

**Published:** 2025-09-26

**Authors:** Xin Yi, Chongshuang Yang, Peng Wang, Razif Abas, Raja Abdul Wafy Raja Muhammad Rooshdi, Jie Yan, Canzhang Liu, Ummi Nadira Daut

**Affiliations:** a Department of Internal Medicine, Faculty of Medicine and Health Sciences, Universiti Putra Malaysia, Serdang, Selangor, Malaysia; b Department 1 of Cardiovasology, North China University of Science and Technology Affiliated Hospital, Tangshan City, Hebei Province, China; c Department of Radiology, Faculty of Medicine and Health Sciences, Universiti Putra Malaysia, Serdang, Selangor, Malaysia; d Department of Surgery, Faculty of Medicine and Health Sciences, Universiti Putra Malaysia, Serdang, Selangor, Malaysia; e Department of Human Anatomy, Faculty of Medicine and Health Sciences, Universiti Putra Malaysia, Serdang, Selangor, Malaysia.

**Keywords:** atherogenic index of plasma, elderly, NHANES, obstructive sleep apnea, STOP-Bang

## Abstract

This study aimed to investigate the association between the atherogenic index of plasma (AIP) and the high risk of obstructive sleep apnea (OSA) in aged person over 60 years, utilizing data from the National Health and Nutrition Examination Survey 2017 to 2020. A cross-sectional analysis was performed on 903 elderly participants from National Health and Nutrition Examination Survey. OSA risk was assessed using a modified STOP-Bang questionnaire. AIP was calculated as log10 (triglycerides/high-density lipoprotein cholesterol). Multivariable logistic regression models adjusted for demographics, lifestyle, and comorbidities were used to evaluate the AIP–OSA association. Subgroup analyses explored effect modifications by age, race, smoking, alcohol use, diabetes, and education. Sensitivity analyses, including quartile-based trend tests, were performed to assess the robustness of the findings. Restricted cubic splines explored potential nonlinear associations between AIP and OSA. Compared to the non-OSA High Risk Group (n = 533), the OSA High Risk group (n = 370) had significantly higher AIP (*P* < .05). In fully adjusted models, each 1-unit AIP increase raised OSA risk by 87% (OR = 1.87, 95% CI: 1.17–3.00, *P* = .0097). Subgroup analyses revealed stronger associations in individuals aged < 70 (OR = 2.50, *P* = .0045), alcohol consumers (OR = 2.21, *P* = .0019), non-diabetics (OR = 2.37, *P* = .0027), and non-Hispanic Black participants (OR = 5.93, *P* = .0014). Restricted cubic splines confirmed a linear relationship (*P*-overall = .0269, *P*-nonlinear = .471). Sensitivity analyses further supported the robustness of these findings. Elevated AIP is independently associated with a higher risk of OSA in older adults. AIP may serve as a potential biomarker for OSA risk stratification in this population.

## 1. Introduction

Obstructive sleep apnea (OSA) represents a growing global health concern, particularly among aging populations. Recent epidemiological data reveal that approximately 40% of older adults meet diagnostic criteria for this condition, with prevalence rates climbing above 80% in specific high-risk groups such as obese individuals or those with metabolic abnormalities.^[1,2]^ The clinical significance of OSA extends beyond sleep disturbances, as it demonstrates robust associations with major health consequences including stroke, coronary artery disease,^[3]^ and type 2 diabetes mellitus,^[4]^ all of which contribute substantially to morbidity in older individuals.

Current diagnostic approaches for OSA in geriatric patients encounter multiple barriers. The frequent absence of classic symptoms, combined with complex medical histories and technical limitations of existing diagnostic tools, creates significant identification challenges.^[5]^ Although polysomnography maintains its position as the definitive diagnostic method,^[6]^ practical constraints including patient discomfort and interference from comorbid conditions often limit its effectiveness in clinical practice.^[7]^ While screening instruments like the STOP-BANG questionnaire have gained popularity,^[8,9]^ their dependence on subjective reporting and limited accuracy in elderly populations highlight the urgent need for more reliable screening approaches.^[10]^ This diagnostic gap underscores the importance of developing novel, objective indicators for OSA detection in older adults.

Atherosclerotic Index of Plasma (AIP) is a biochemical marker closely associated with lipid metabolism pathways,^[11]^ and is derived by logarithmic transformation of triglycerides (TG) and high-density lipoprotein cholesterol (HDL-C).^[12]^ Substantial evidence connects elevated AIP values with increased cardiovascular risk^[13,14]^ and various metabolic disturbances.^[15]^ AIP levels are associated with metabolic abnormalities,^[16]^ and OSA is linked to similar issues, such as obesity, insulin resistance, and dyslipidemia.^[17]^ These conditions may be worsened by OSA through mechanisms like systemic inflammation, oxidative stress, and endothelial dysfunction,^[18]^ which are also associated with elevated AIP levels.^[19]^ This suggests a potential connection between AIP and OSA.

Given that both AIP and OSA are associated with systemic metabolic disturbances, it is plausible that elevated AIP may serve as an indicator of increased OSA risk, especially in aging populations where both conditions are prevalent. Understanding this connection could improve early identification and risk stratification for OSA, enabling more targeted interventions in at-risk individuals. Additionally, given that AIP is a simple, reproducible, and cost-effective biomarker, it may offer a practical means of identifying individuals at heightened risk for OSA in clinical settings.

To address this knowledge gap, we analyzed data from the 2017 to 2020 National Health and Nutrition Examination Survey (NHANES) to investigate the relationship between AIP and OSA risk in adults, with particular attention to potential dose–response patterns. This examination may provide valuable insights for developing improved screening strategies for OSA detection in aging populations.

## 2. Methods and materials

### 2.1. Study design and population

The NHANES is a nationally representative, complex, multistage probability survey that evaluates health and nutritional status among civilians in the U.S. who are not institutionalized. Conducted by the CDC’s National Center for Health Statistics, the survey follows strict ethical protocols, including approval by the NCHS Institutional Review Board. The study was conducted in accordance with the ethical standards laid down in the Declaration of Helsinki and its later amendments. All enrolled individuals submitted written consent before participation. Since this study utilized publicly available, anonymized NHANES data (2017–2020), no additional ethics approval was required under institutional policies. Clinical trail number: not applicable. To minimize potential sources of bias, we used nationally representative data from NHANES 2011 to 2018, applied clear inclusion and exclusion criteria to ensure data completeness and quality, and conducted subgroup analyses by sex and age to assess the consistency of associations across different population groups.

**Figure 1.**
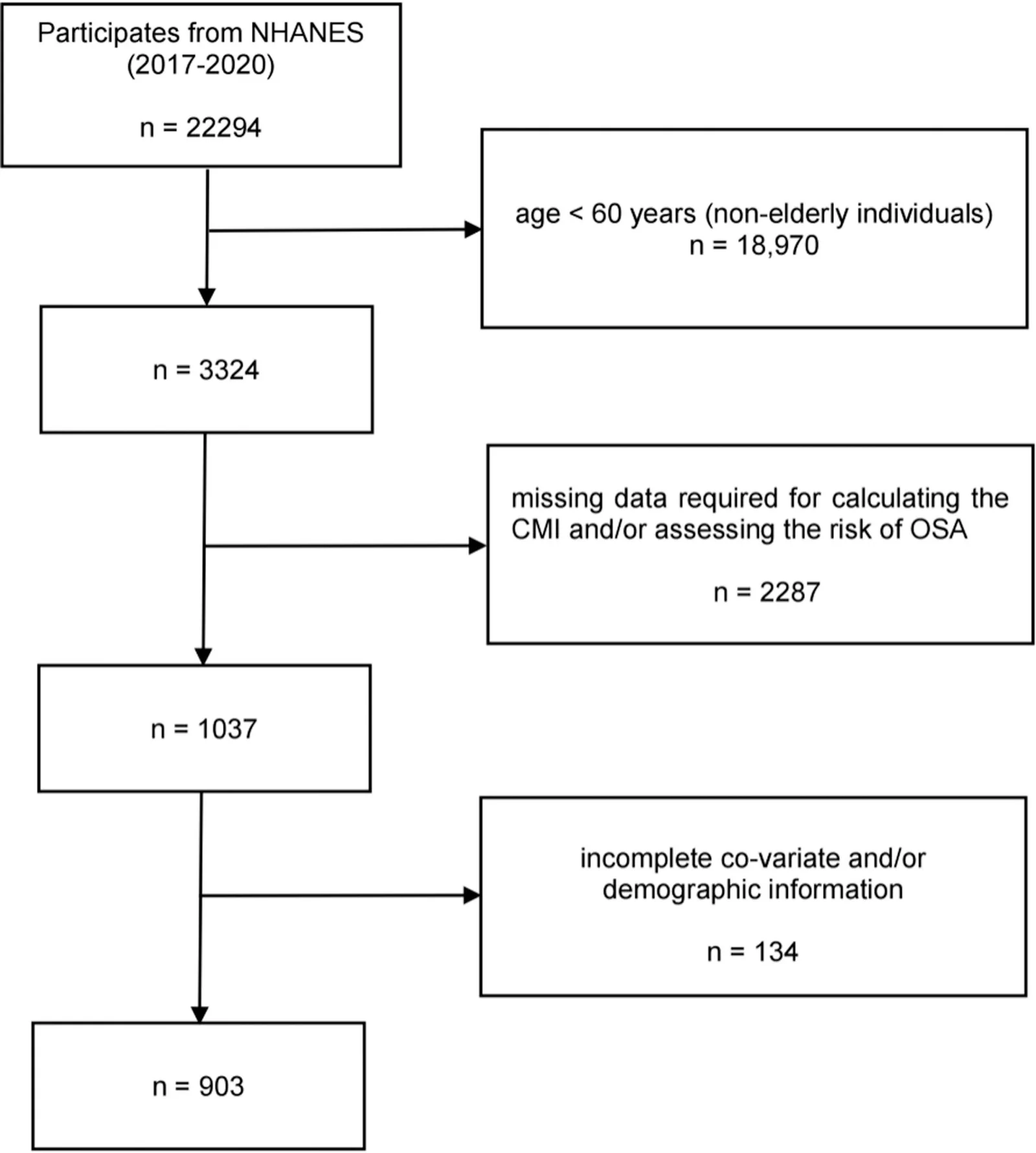
Participants screening process.

From an initial pool of 22,294 participants, we excluded individuals aged < 60 years (n = 15,391), those lacking data for AIP calculation or OSA risk assessment (n = 5732), and participants with incomplete covariate information (n = 268). The final analytical cohort comprised 903 elderly adults (aged ≥ 60 years) with complete demographic, clinical, and laboratory data. A detailed enrollment flowchart is provided in Figure 1.

### 2.2. OSA High Risk assessment

In this study, the risk of OSA was assessed using a modified version of the STOP-Bang questionnaire.^[20,21]^ Due to the absence of certain items, such as neck circumference, in the NHANES dataset, 7 components were included for scoring, each assigned 0 or 1 point, resulting in a total score ranging from 0 to 7. The components were defined as follows: snoring (S) was scored 1 if participants reported frequent snoring (≥5 nights per week) over the past 12 months; in cases of uncertainty, family members or cohabitants were consulted for confirmation. Tiredness (T) was scored 1 if participants reported experiencing daytime fatigue or sleepiness “almost always” (16–30 times per month) within the past year. Observed apnea (O), traditionally defined as others witnessing cessation of breathing during sleep, was assessed through participant self-report combined with confirmation by family or cohabitants, encompassing snorting, gasping, or apnea occurring at least 1 to 2 nights per week; this approach may differ from the classical definition and should be interpreted with caution. Hypertension (P) was assigned 1 point if average systolic blood pressure (BP) was ≥ 130 mm Hg or diastolic BP ≥ 80 mm Hg. Body mass index (BMI) (B) was scored 1 for values ≥ 35 kg/m². Age (A) was scored 1 for individuals aged ≥ 50 years, and gender (G) was assigned 1 point for males. Following established STOP-BANG recommendations,^[22]^ a total score of 3 or higher was used to classify participants as high risk for OSA. The participants were then divided into 2 groups: the non-OSA High Risk group (n = 533) and the OSA High Risk group (n = 370).

All BP measurements were conducted at the Mobile Examination Center. Arm circumference was measured first to determine the appropriate cuff size, as outlined in the Anthropometric Procedures Manual. After the participant rested for 5 minutes, blood pressure was measured 3 times at 60-second intervals using the Omron HEM-907XL digital upper arm monitor (Omron Healthcare Co., Ltd., Kyoto, Japan). Blood pressure was measured on the right arm unless contraindicated. The average of the 3 measurements for systolic BP and diastolic BP was used as the participant’s BP level. BMI was calculated by dividing the weight (kg) by the square of the height (m). More detailed information is available on the NHANES website.

### 2.3. AIP calculation method

The AIP is calculated using the formula:


$$\begin{array}{l} AIP=\log\left(\frac{TG}{HDL-C}\right) \end{array}$$

where TG and HDL-C are measured in mmol/L.

TG levels were quantified enzymatically (Wahlefeld method; Roche (2014)). In this assay, lipoprotein lipase hydrolyzes TGs to release glycerol, which is subsequently oxidized to yield dihydroxyacetone phosphate and hydrogen peroxide. The peroxide then reacts with 4-aminophenazone and 4-chlorophenol catalyzed by peroxidase, generating a red chromophore whose intensity correlates with TG content. For HDL-C measurement, a third-generation assay was employed. Non-HDL cholesterol was precipitated using magnesium/dextran sulfate, leaving HDL cholesterol in solution. The retained HDL-C was enzymatically converted to free cholesterol via PEG–cholesterol esterase, followed by oxidation with PEG–cholesterol oxidase to produce hydrogen peroxide. This peroxide mediated the reaction of 4-amino-antipyrine with HSDA, forming a purple/blue product quantified spectrophotometrically at 600 nm. These methods, as outlined by NHANES, were used to measure TG and HDL-C for AIP calculation in this study.

### 2.4. Covariates measurement

The analysis accounted for key demographic variables: age, race, education levels, and poverty-income ratio (PIR). Racial/ethnic groups included Mexican American, non-Hispanic White, non-Hispanic Black, other Hispanic, and other races. Education levels were stratified into 5 categories: <9th grade, 9 to 11th grade (including 12th grade without diploma), high school graduate/GED, some college/AA degree, and college graduate or higher. Additional covariates included alcohol use, current smoking status, and diabetes diagnosis. Variables such as gender, BMI, and hypertension were initially evaluated but omitted from the final model to avoid multicollinearity, as they were already incorporated into the OSA risk assessment framework. This exclusion strengthened the model’ s validity by preventing overlap in predictive factors.

### 2.5. Definition of main and secondary outcomes

The main outcome of this study was to assess the association between the AIP and the high risk of OSA. We hypothesized that higher AIP levels are associated with increased OSA risk. This was evaluated using multivariate logistic regression models, adjusted for demographic, behavioral, and clinical factors, to determine AIP’s independent predictive value for OSA.

The secondary outcome was to examine the influence of factors such as age, alcohol use, education, and diabetes status on the AIP–OSA relationship through subgroup analysis, and to explore potential nonlinear associations between AIP and OSA risk using restricted cubic spline (RCS) regression analysis. Additionally, sensitivity analyses, including quartile analysis and trend tests, were conducted to assess the robustness of the results.

### 2.6. Statistical analysis

Data analysis was performed using R software (version 4.5.0, R Foundation for Statistical Computing, Vienna, Austria). Normality of variables was assessed by the Kolmogorov–Smirnov test. Normally distributed data were presented as mean ± standard deviation and compared between groups using the independent samples *t* test; non-normally distributed data were expressed as median (interquartile range) and compared using the Mann–Whitney *U* test. Multivariable logistic regression models were constructed to examine the association between AIP levels and high risk of OSA. Three models were developed: Model 1 (unadjusted), Model 2 (adjusted for age and race), and Model 3 (fully adjusted for age, race, education level, income-to-poverty ratio, alcohol consumption, recent smoking, and diabetes). Additionally, subgroup analyses were performed based on factors such as age, race, smoking status, alcohol consumption, diabetes, and education level to further explore the relationship between AIP and high risk of OSA in these specific groups. RCS curves were used to explore potential nonlinear relationships between AIP and high risk of OSA. AIP was also categorized into quartiles for sensitivity analyses within the fully adjusted model. Trend tests were conducted to assess dose–response relationships. Statistical significance was set at *P* < .05.

## 3. Results

### 3.1. The baseline characteristics of all participants

The study included 903 participants aged 60 to 80 years (498 males, 405 females), stratified into 2 groups: non-OSA High Risk (n = 533) and OSA High Risk (n = 370). Comparative analysis revealed significant between-group differences (*P* < .05) in several parameters. The OSA High Risk group demonstrated elevated anthropometric measures (height, weight, and BMI) and AIP values compared to their counterparts. This group also contained a greater proportion of male participants, non-Hispanic Black individuals, and diabetic patients. In contrast, HDL-C levels were markedly lower in the OSA High Risk cohort. No other variables showed statistically significant intergroup differences (*P* > .05). As shown in Table 1.

**Table 1:** Baseline characteristics of participants.

Variable	Over all (n = 903)	Non-OSA High Risk (n = 533)	OSA High Risk (n = 370)	*P*
Continuous variables, median (Q1–Q3) or mean ± SD				
Age (yr)	68 (61, 74)	68 (63, 74)	68 (63, 75)	.371
Income-to-poverty ratio	2.43 (1.425, 4.35)	2.57 (1.48, 4.6175)	2.37 (1.39, 4.2)	.343
Height (cm²)	166.19 ± 9.62	163.02 ± 9.38	168.39 ± 9.161	**<.001**
Weight (kg)	80.70 (68.45, 93.60)	73.7 (64.9, 84.08)	85.40 (73.2, 100.9)	**<.001**
BMI (kg/m²)	28.70 (25.40, 33.40)	28.05 (25.20, 30.90)	29.5 (25.06, 35.40)	**<.001**
TG (mmol/L)	1.118 (0.78, 1.51)	1.095 (0.76, 1.52)	1.14 (0.80, 1.50)	.317
HDL-C (mmol/L)	1.34 (1.11, 1.63)	1.435 (1.16. 1.73)	1.29 (1.09, 1.55)	**<.001**
AIP	-0.09 (-0.30, 0.10)	-0.12 (-0.33, 0.08)	-0.07 (-0.26, 0.12)	**.003**
Categorical variables, (n)				
Gender				
Male	498	116	382	**<.001**
Female	405	254	151	
Race				
Mexican American	79	34	45	**.036**
Other Hispanic	97	44	53	
Non-Hispanic White	386	164	222	
Non-Hispanic Black	233	76	157	
Other Races	108	52	56	
Education level				
Level 1	69	28	41	.630
Level 2	102	35	67	
Level 3	241	97	144	
Level 4	280	120	160	
Level 5	211	90	121	
Alcohol consumption				
Yes	823	332	491	.261
No	80	38	42	
Recent smoking				
Yes	135	55	80	1.000
No	768	315	453	
Diabetes				
Yes	254	86	168	**.008**
No	649	284	365	

Level 1: <9th grade; level 2: 9–11th grade (includes 12th grade with no diploma); level 3: high school graduate/GED or equivalent; level 4: some college or AA degree; level 5: college graduate or above. Bold values indicate statistical significance (*P* < .05).

AIP = atherosclerotic index of plasma; HDL = high-density lipoprotein cholesterol; TG = triglycerides.

### 3.2. Multivariate logistic regression analysis of AIP and OSA risk

**Figure 2.**
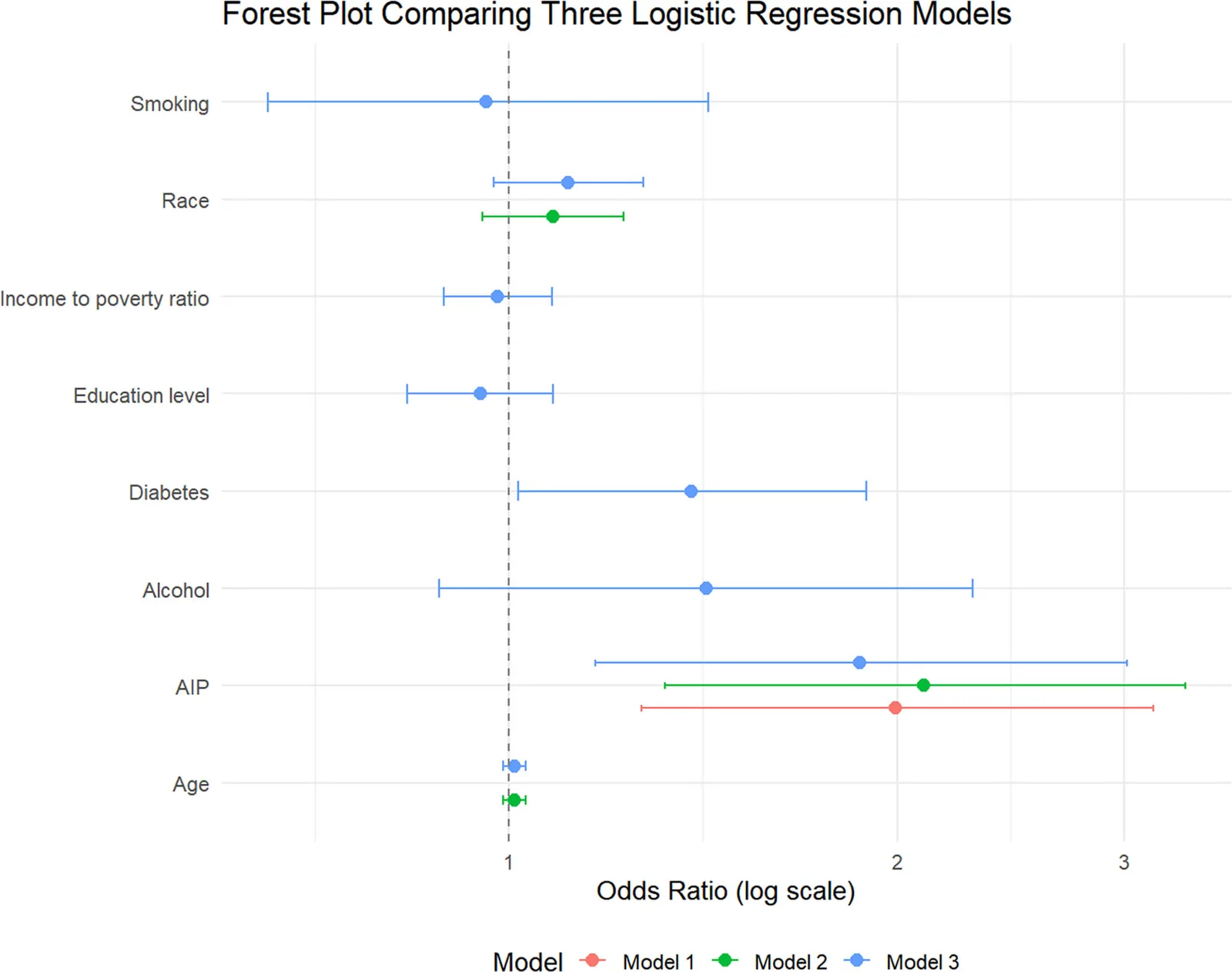
Forest plot comparing 3 logistic regression models.

Three progressively adjusted logistic regression models were constructed to evaluate the AIP–OSA association. The unadjusted Model 1 revealed a strong positive relationship (OR = 2.00, *P* = .003), with each unit AIP increment corresponding to doubled OSA risk. This association persisted in Model 2 after demographic adjustment (age, race), showing even greater effect magnitude (OR = 2.10, *P* = .00176) representing 110% elevated risk. The fully adjusted Model 3, incorporating behavioral and socioeconomic factors (smoking, diabetes, alcohol use, education, and income), maintained statistical significance (OR = 1.87, *P* = .00970), translating to an 87% risk elevation per AIP unit. Notably, diabetes emerged as an independent predictor (OR = 1.39, *P* = .040), associated with 39% higher OSA likelihood. As shown in Table 2 and Figure 2.

**Table 2:** Three multivariate logistic regression models.

Variable	OR (95% CI)	Std error	Z	*P*
Model 1 (unadjusted)				
AIP	2.00 (1.27–3.16)	0.233	2.96	**.003**
Model 2 (adjusted for age and race)				
AIP	2.10 (1.32–3.35)	0.237	3.13	**.002**
Age (yr)	1.01 (0.99–1.03)	0.0102	0.926	.355
Race	1.08 (0.95–1.23)	0.0644	1.22	.223
Model 3 (fully adjusted) Including age, race, education level, income-to-poverty ratio, alcohol consumption, recent smoking, and diabetes				
AIP	1.87 (1.17–3.02)	0.242	2.59	**.010**
Age (yr)	1.01 (0.99–1.03)	0.0104	0.838	.402
Race	1.11 (0.97–1.27)	0.0683	1.55	.122
Recent smoking	0.96 (0.65–1.43)	0.200	-0.203	.839
Diabetes	1.39 (1.02–1.89)	0.159	2.05	**.040**
Alcohol consumption	1.42 (0.88–2.29)	0.243	1.45	.147
Education level	0.95 (0.83–1.08)	0.0666	-0.781	.435
Income-to-poverty ratio	0.98 (0.89–1.08)	0.0492	-0.418	.676

Bold values indicate statistical significance (*P* < .05).

AIP = atherosclerotic index of plasma.

### 3.3. Subgroup analysis

**Figure 3.**
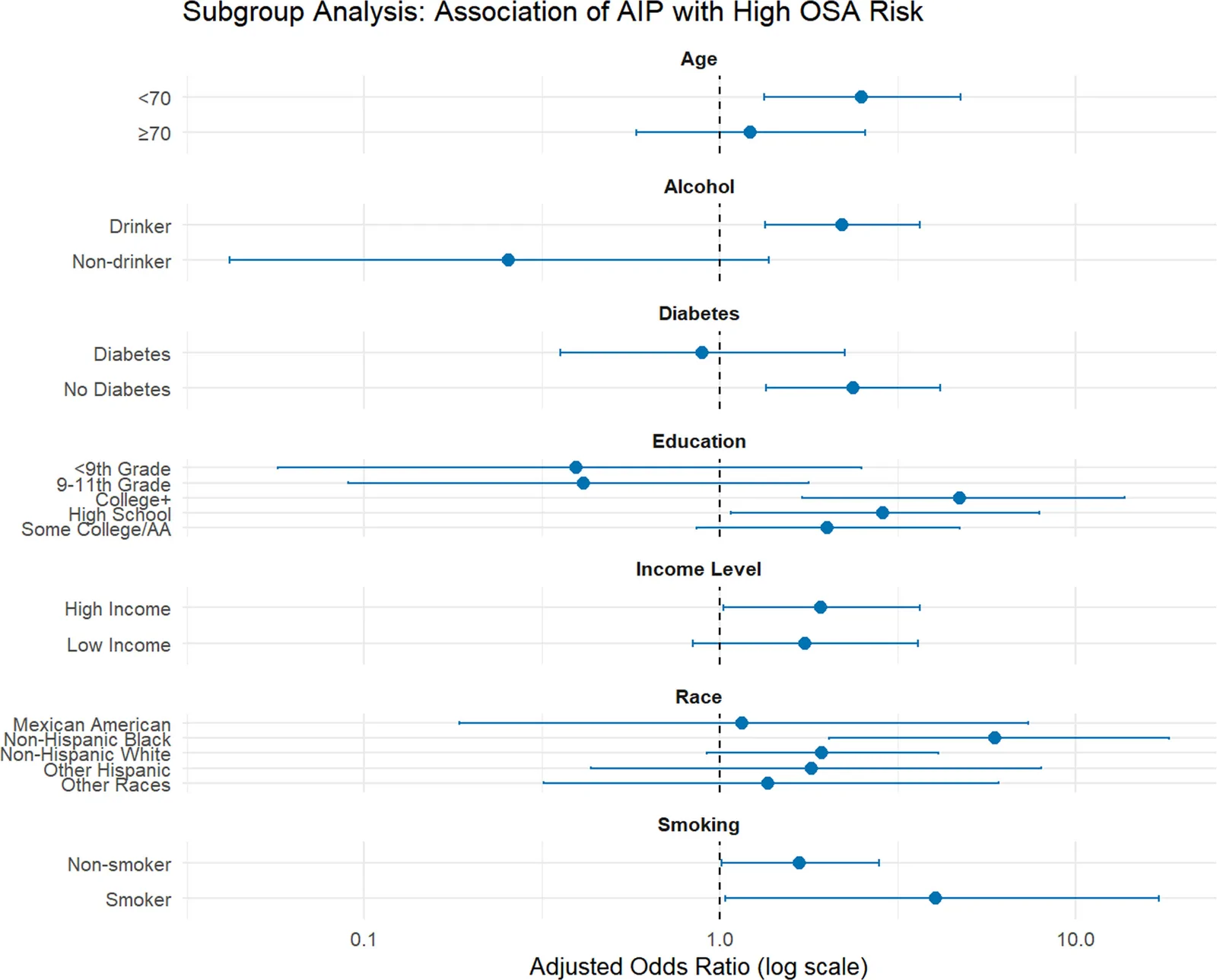
Forest plot for subgroup analysis.

Prior to analysis, continuous variables were dichotomized with age stratified as < 70 versus ≥ 70 years and income categorized at the median into low and high groups. The age-stratified analysis revealed a marked differential effect, with participants under 70 years displaying a robust association between elevated AIP and OSA risk (adjusted OR: 2.50, 95% CI: 1.34–4.75; *P* = .0045), while no such relationship reached statistical significance in older participants (*P* = .6005). Alcohol consumption status emerged as an important effect modifier, with drinkers demonstrating a strong positive association (OR: 2.21, 1.35–3.66; *P* = .0019) that was absent in nondrinkers (*P* = .1213). Diabetes status significantly influenced the observed relationships. Non-diabetic individuals showed a clear dose–response relationship (OR: 2.37, 1.35–4.18; *P* = .0027), whereas diabetic participants exhibited no statistically meaningful association (*P* = .8115). Educational attainment similarly modified the effect size, with progressively stronger associations observed from high school graduates (OR: 2.87, 1.08–7.95; *P* = .0378) to college-educated individuals (OR: 4.73, 1.71–13.77; *P* = .0034), though no significant effects were detected in less educated subgroups. Socioeconomic factors demonstrated variable associations, with significant findings limited to higher income participants (OR: 1.93, 1.03–3.65; *P* = .0425). Racial disparities were particularly notable, with non-Hispanic Black individuals showing the strongest association (OR: 5.93, 2.04–18.35; *P* = .0014) among all demographic groups analyzed. Tobacco use status maintained significant associations in both smokers (OR: 4.05, 1.04–17.20; *P* = .0492) and nonsmokers (*P* = .0462), though with markedly different effect magnitudes. As shown in Table 3 and Figure 3.

**Table 3:** Subgroup analysis.

Variable	OR (95% CI)	Std error	Z	*P*
Model 3 (fully adjusted) Including age, race, education level, income-to-poverty ratio, alcohol consumption, recent smoking, and diabetes				
Age				
<70	2.50 (1.34, 4.75)	0.32	2.84	**.005**
≥70	1.22 (0.58, 2.56)	0.38	0.52	.601
Alcohol consumption				
No	0.25 (0.04, 1.38)	0.88	-1.55	.121
Yes	2.21 (1.35, 3.66)	0.25	3.11	**.002**
Diabetes				
No	2.37 (1.35, 4.18)	0.29	2.99	**.003**
Yes	0.89 (0.36, 2.24)	0.47	-0.24	.812
Education				
Level 1	0.39 (0.06, 2.50)	0.95	-0.98	.328
Level 2	0.41 (0.09, 1.78)	0.75	-1.17	.241
Level 3	2.87 (1.08, 7.95)	0.51	2.08	**.038**
Level 4	2.00 (0.86, 4.73)	0.43	1.60	.109
Level 5	4.73 (1.71, 13.77)	0.53	2.93	**.003**
Income-to-poverty ratio				
High	1.93 (1.03, 3.65)	0.32	2.03	**.043**
Low	1.73 (0.84, 3.62)	0.37	1.48	.139
Race				
Mexican American	1.16 (0.19, 7.37)	0.93	0.16	.877
Other Hispanic	1.81 (0.43, 8.01)	0.74	0.80	.421
Non-Hispanic White	1.94 (0.92, 4.12)	0.38	1.73	.083
Non-Hispanic Black	5.93 (2.04, 18.35)	0.56	3.19	**.001**
Other Races	1.37 (0.32, 6.07)	0.75	0.42	.673
Recent smoking				
No	1.68 (1.01, 2.80)	0.26	1.99	**.046**
Yes	4.05 (1.04, 17.20)	0.71	1.97	**.049**

Level 1: <9th grade; level 2: 9–11th grade (includes 12th grade with no diploma); level 3: high school graduate/GED or equivalent; level 4: some college or AA degree; level 5: college graduate or above. Bold values indicate statistical significance (*P* < .05).

AIP = atherosclerotic index of plasma.

### 3.4. RCS regression analysis of AIP and OSA risk

**Figure 4.**
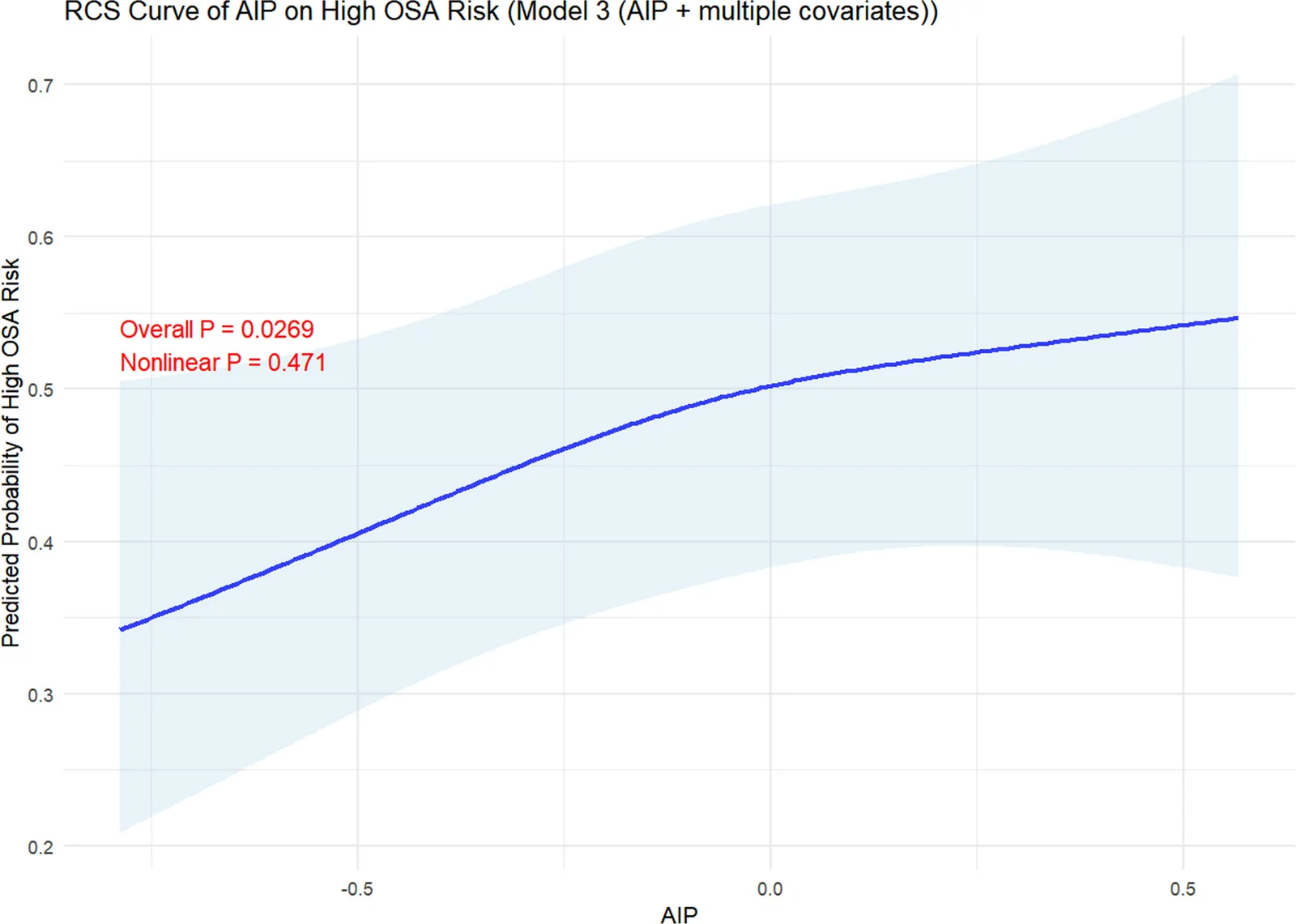
RCS curve of AIP and OSA high risk in model 3. AIP = atherosclerotic index of plasma, OSA = obstructive sleep apnea, RCS = restricted cubic spline.

The dose–response relationship between AIP and OSA risk was evaluated using RCS regression. After comprehensive adjustment for demographic and clinical confounders in Model 3 (including age, race, smoking status, diabetes, hypertension, education level, and income), the analysis revealed a statistically significant association (*P*-overall = .027). This confirms AIP as an independent predictor of OSA risk. The non-linearity test was non-significant (*P*-nonlinear = .471), supporting a linear rather than curvilinear relationship across the AIP spectrum. Visual inspection of the RCS plot corroborated these analytical findings, demonstrating a consistent linear trend between increasing AIP values and elevated OSA risk. The graphical representation confirms that the association remains stable regardless of additional covariate adjustment. As shown in Figure 4.

### 3.5. Sensitivity analysis

**Figure 5.**
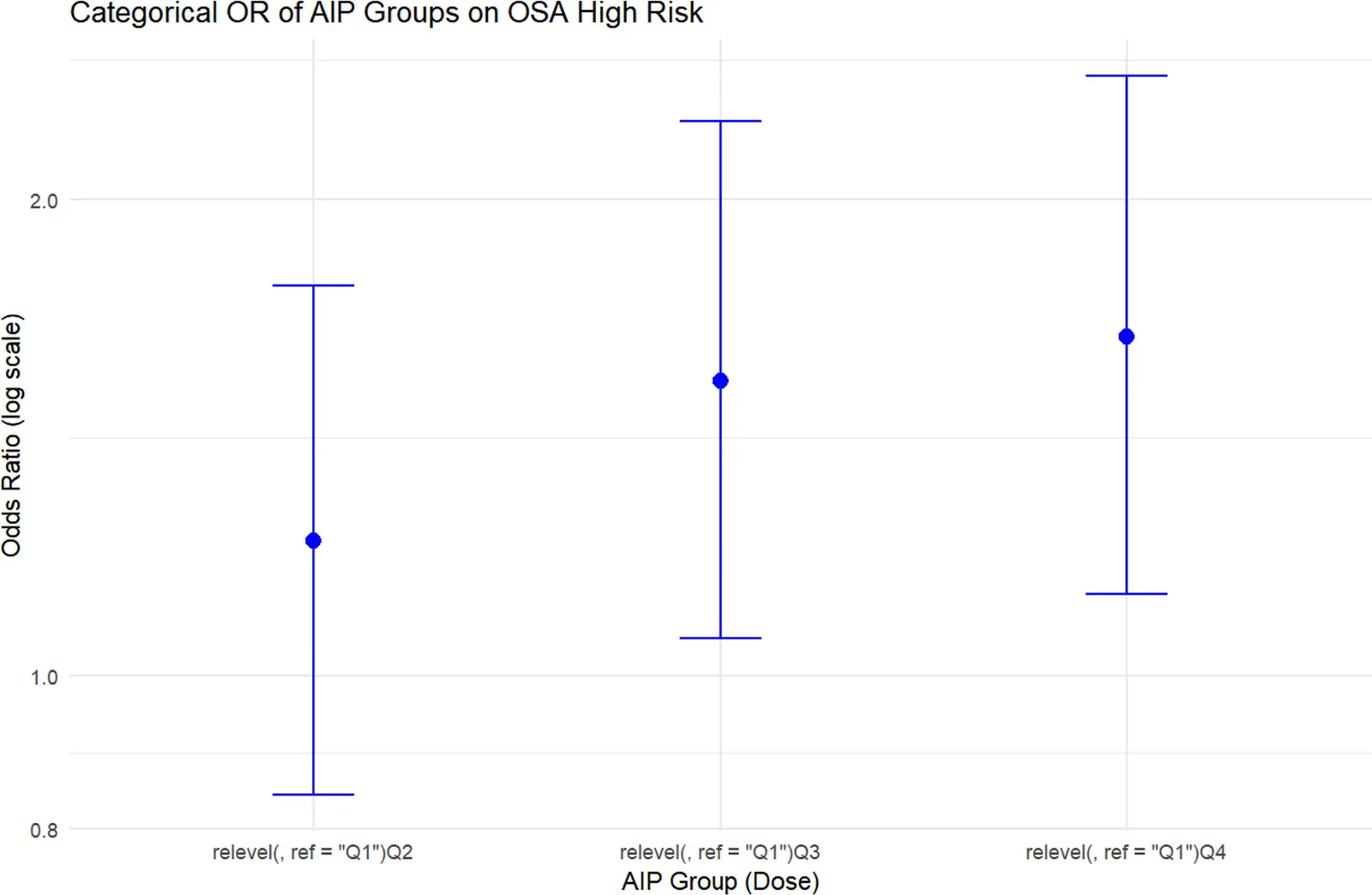
Categorical OR of AIP on OSA high risk. AIP = atherosclerotic index of plasma, OSA = obstructive sleep apnea.

To assess the robustness and consistency of the association between AIP and OSA risk, we conducted sensitivity analyses using both quartile stratification and linear trend testing. In the quartile analysis, AIP was divided into 4 categories, and associations were evaluated using the fully adjusted Model 3. Compared with the lowest quartile (Q1), higher AIP levels were associated with an increased risk of OSA: Q2 (OR = 1.18, *P* = .387), Q3 (OR = 1.50, *P* = .0361), and Q4 (OR = 1.53, *P* = .0314). This pattern suggests a graded elevation in OSA risk across ascending AIP quartiles, with statistically significant associations observed in the upper 2 quartiles. In addition, a linear trend analysis revealed a significant positive trend between AIP and OSA risk (β = 1.17, SE = 0.0629, *P* = .0144), indicating a progressive increase in OSA susceptibility with rising AIP levels. Together, these findings support a consistent and dose-related association between AIP and OSA risk, reinforcing the potential utility of AIP as a clinical biomarker for risk stratification. As shown in Table 4 and Figure 5.

**Table 4:** AIP quartile analysis.

Variable	OR (95% CI)	Std error	Z	*P*
Model 3 (fully adjusted) Including age, race, education level, income-to-poverty ratio, alcohol consumption, recent smoking, and diabetes				
AIP Quartile				
Q1	Reference	Reference	Reference	Reference
Q2	1.18 (0.810–1.72)	0.193	0.86	.387
Q3	1.50 (1.03–2.20)	0.194	2.10	**.036**
Q4	1.53 (1.04–2.27)	0.199	2.15	**.031**

Bold values indicate statistical significance (*P* < .05).

AIP = atherosclerotic index of plasma.

## 4. Discussion

In this nationally representative cross-sectional study of individuals aged 60 years and older, we first analyzed baseline data and found that elderly individuals at high risk for OSA, identified via a simplified STOP-BANG screening tool, exhibited lower HDL-C levels and a higher prevalence of diabetes. This suggests that beyond the known factors incorporated in the STOP-BANG scale (such as sex, hypertension, and BMI) lipid and metabolic parameters may play a crucial role in the pathophysiology of OSA onset and progression. These findings align well with previous studies.^[23–25]^ However, unlike some prior reports,^[26]^ we did not observe a statistically significant difference in TG levels between the non-OSA High Risk and OSA High Risk group. This discrepancy may be attributed to age-related metabolic changes, wherein the influence of OSA on TG levels becomes less pronounced in older adults. It also implies that single lipid parameters may inadequately capture the complexity of systemic lipid metabolism, highlighting the potential superiority and stability of composite indices in this context. Furthermore, we observed significant racial disparities between these 2 groups, with a notable increase in non-Hispanic Black individuals among the high-risk elderly population. This observation is consistent with prior research,^[27]^ which attributes elevated OSA risk in this group to multifactorial influences, including anatomical variations,^[28]^ metabolic health status,^[29]^ and sleep behaviors.^[30]^

Building upon these observations, our study systematically examined the association between AIP and high risk of OSA. Previous study have indicated that individual lipid parameters, such as TG, HDL, LDL, and TC, show no significant correlation with OSA.^[31]^ However, AIP, which reflects the balance between triglycerides and HDL cholesterol, offers a more comprehensive measure of lipid metabolism than these individual lipid markers.^[32,33]^ Our findings from multivariate logistic regression analyses, after adjusting for multiple confounders, demonstrated that each unit increase in AIP was associated with a 1.9-fold higher odds of being classified as high risk for OSA. A previous study in a cohort with an average age of 50 years identified elevated AIP as one of the risk factors for OSA.^[34]^ These findings are consistent with our results, where an elderly cohort with an average age of 68 years also exhibited a strong association between elevated AIP and increased OSA risk. This suggests that AIP can be considered an independent risk factor for high-risk OSA in older adults. This consistency across age groups highlights the robustness of AIP as a potential marker for OSA risk, emphasizing the need for its further investigation in diverse populations. The elevation of AIP likely mirrors underlying metabolic disturbances that increase elderly individuals’ susceptibility to high risk of OSA via mechanisms such as dyslipidemia, systemic inflammation, and insulin resistance.^[35,36]^ Elevated AIP may lead to endothelial dysfunction and atherosclerosis,^[37]^ both of which have been implicated in the pathogenesis of OSA.^[38,39]^ In addition to metabolic factors, age-related changes including diminished pharyngeal muscle activity,^[40]^ altered pharyngeal muscular dilatory reflexes,^[41]^ structural modifications of the upper airway leading to increased airway resistance during sleep,^[42–44]^ and reduced pulmonary elastic recoil further contribute to OSA pathogenesis in older adults.^[45]^ These factors may form a vicious cycle, exacerbating OSA risk. These age-related physiological changes, combined with metabolic dysregulation, may result in a synergistic effect that accelerates the onset and progression of OSA in the elderly. Additionally, the reduced ability of older individuals to adapt to sleep-related oxygen desaturation could further amplify the severity of OSA, creating a vicious cycle that exacerbates both metabolic disturbances and OSA severity. The sensitivity analyses reaffirmed the association between AIP and OSA risk, indicating that this relationship persists across different strata of lipid profiles. These findings underscore the robustness of the primary results and point to the potential utility of AIP as a clinically relevant marker, even in individuals with only mild dyslipidemia.

Subgroup analyses underscored the heterogeneity in the association between AIP and OSA risk, influenced by age, metabolic integrity, and social determinants. This association was pronounced exclusively in individuals under 70 years of age, suggesting that lipid-related indices such as AIP may better capture metabolically driven OSA risk in younger populations. Previous studies have indicated that lipid metabolism undergoes significant alterations with advancing age.^[46]^ In contrast, among individuals aged 70 years and older, age-related anatomical and neuromuscular changes, compounded by the cumulative burden of comorbidities, may overshadow metabolic factors, thereby attenuating the relevance of dyslipidemia in the pathogenesis of OSA. No significant association was observed in diabetic patients, which may reflect the complex metabolic milieu inherent to this condition. Chronic hyperglycemia, insulin resistance, and the widespread use of lipid-lowering therapies could confound lipid profiles,^[47,48]^ thus diminishing the discriminative power of AIP within this subgroup. Conversely, the strengthened associations identified in alcohol consumers and smokers may be attributed to the pro-inflammatory and pro-atherogenic synergism of these exposures, which exacerbate metabolic derangements contributing to OSA development.^[49]^ Sociodemographic factors, including educational attainment, income, and ethnicity, further modulated the strength of these associations. Higher effect sizes were observed among individuals with higher education and income levels, likely reflecting disparities in healthcare access and diagnostic precision. In Hispanic Black populations, the significant association between AIP and elevated OSA risk may be attributed to a confluence of biological susceptibilities and socio-environmental factors that predispose this group to higher OSA prevalence.^[50]^

Several limitations should be noted in this research. Firstly, the cross-sectional nature of the study precludes definitive conclusions regarding causality between AIP and OSA risk. Secondly, the absence of key metrics such as neck circumference necessitated the use of an adjusted STOP-BANG questionnaire, which may compromise diagnostic accuracy and introduce potential misclassification errors. Furthermore, reliance on self-reported sleep parameters could lead to recall bias and inter-subject variability, potentially influencing result reliability. Therefore, future investigations involving large-scale, multicenter prospective cohorts are needed to verify these findings and clarify this relationship more thoroughly.

In conclusion, our findings suggest a significant association between elevated AIP and an increased risk of OSA in the elderly population. Given that AIP is an easily accessible and cost-effective lipid-based biomarker, it holds promise as a practical tool for early screening and risk stratification of OSA in older adults. Further longitudinal and mechanistic investigations are essential to confirm these associations and to explore potential intervention strategies.

## Acknowledgments

We would like to acknowledge NHANES for making the publicly available data accessible for use in this study.

## Author contributions

**Conceptualization:** Xin Yi.

**Data curation:** Xin Yi, Jie Yan, Canzhang Liu.

**Formal analysis:** Xin Yi, Chongshuang Yang.

**Investigation:** Xin Yi.

**Methodology:** Xin Yi, Chongshuang Yang, Peng Wang.

**Software:** Xin Yi.

**Supervision:** Ummi Nadira Daut

**Writing – original draft:** Xin Yi, Razif Abas.

**Writing – review & editing:** Raja Abdul Wafy Raja Muhammad Rooshdi, Ummi Nadira Daut.

## References

[1] Canever JB Zurman G Vogel F. Worldwide prevalence of sleep problems in community-dwelling older adults: A systematic review and meta-analysis. Sleep Med. 2024;119:118–34. 10.1016/j.sleep.2024.03.040 38669835

[2] Ghavami T Kazeminia M Ahmadi N Rajati F. Global prevalence of obstructive sleep apnea in the elderly and related factors: a systematic review and meta-analysis study. J Perianesthesia Nurs. 2023;38:865–75. 10.1016/j.jopan.2023.01.018 37318436

[3] Senaratna CV Perret JL Lodge CJ. Prevalence of obstructive sleep apnea in the general population: a systematic review. Sleep Med Rev. 2017;34:70–81. 10.1016/j.smrv.2016.07.002 27568340

[4] Brown J Yazdi F Jodari-Karimi M Owen JG Reisin E. Obstructive sleep apnea and hypertension: updates to a critical relationship. Curr Hypertens Rep. 2022;24:173–84. 10.1007/s11906-022-01181-w PMC8897114 35246797

[5] Reutrakul S Mokhlesi B. Obstructive sleep apnea and diabetes: a state of the art review. Chest. 2017;152:1070–86. 10.1016/j.chest.2017.05.009 PMC5812754 28527878

[6] Kirk J Wickwire EM Somers VK Johnson DA Albrecht JS. Undiagnosed obstructive sleep apnea increases risk of hospitalization among a racially diverse group of older adults with comorbid cardiovascular disease. J Clin Sleep Med. 2023;19:1175–81. 10.5664/jcsm.10526 PMC10315599 36803353

[7] Osorio RS Martínez-García MA Rapoport DM. Sleep apnoea in the elderly: a great challenge for the future. Eur Respir J. 2021;59:2101649. 10.1183/13993003.01649-2021 PMC8942873 34561285

[8] Gomes T Benedetti A Lafontaine AL Kimoff RJ Robinson A Kaminska M. Validation of STOP, STOP-BANG, STOP-BAG, STOP-B28, and GOAL screening tools for identification of obstructive sleep apnea in patients with Parkinson disease. J Clin Sleep Med. 2023;19:45–54. 10.5664/jcsm.10262 PMC9806789 36004740

[9] Hwang M Zhang K Nagappa M Saripella A Englesakis M Chung F. Validation of the STOP-Bang questionnaire as a screening tool for obstructive sleep apnoea in patients with cardiovascular risk factors: a systematic review and meta-analysis. BMJ Open Respir Res. 2021;8:e000848. 10.1136/bmjresp-2020-000848 PMC7934717 33664122

[10] Iannella G Vicini C Colizza A. Aging effect on sleepiness and apneas severity in patients with obstructive sleep apnea syndrome: a meta-analysis study. Eur Arch Oto-Rhino-Laryngol. 2019;276:3549–56. 10.1007/s00405-019-05616-0 31482333

[11] Min Q Wu Z Yao J. Association between atherogenic index of plasma control level and incident cardiovascular disease in middle-aged and elderly Chinese individuals with abnormal glucose metabolism. Cardiovasc Diabetol. 2024;23:54. 10.1186/s12933-024-02144-y PMC10854096 38331798

[12] Dobiásová M Frohlich J. The plasma parameter log (TG/HDL-C) as an atherogenic index: correlation with lipoprotein particle size and esterification rate in apoB-lipoprotein-depleted plasma (FER(HDL)). Clin Biochem. 2001;34:583–8. 10.1016/s0009-9120(01)00263-6 11738396

[13] Duiyimuhan G Maimaiti N. The association between atherogenic index of plasma and all-cause mortality and cardiovascular disease-specific mortality in hypertension patients: a retrospective cohort study of NHANES. BMC Cardiovasc Disord. 2023;23:452. 10.1186/s12872-023-03451-0 PMC10496369 37697281

[14] Zhang Y Chen S Tian X. Association between cumulative atherogenic index of plasma exposure and risk of myocardial infarction in the general population. Cardiovasc Diabetol. 2023;22:210. 10.1186/s12933-023-01936-y PMC10436658 37592247

[15] Yin B Wu Z Xia Y Xiao S Chen L Li Y. Non-linear association of atherogenic index of plasma with insulin resistance and type 2 diabetes: a cross-sectional study. Cardiovasc Diabetol. 2023;22:157. 10.1186/s12933-023-01886-5 PMC10311747 37386500

[16] Montserrat-de la PS Del Carmen Naranjo M Lopez S. Immediate-release niacin and a monounsaturated fatty acid-rich meal on postprandial inflammation and monocyte characteristics in men with metabolic syndrome. Clin Nutr. 2023;42:2138–50. 10.1016/j.clnu.2023.08.017 37774650

[17] Velescu DR Marc MS Traila D. A Narrative review of self-reported scales to evaluate depression and anxiety symptoms in adult obstructive sleep apnea patients. Medicina (Kaunas). 2024;60:261. 10.3390/medicina60020261 PMC10889932 38399548

[18] Peracaula M Torres D Poyatos P. Endothelial dysfunction and cardiovascular risk in obstructive sleep apnea: a review article. Life (Basel). 2022;12:537. 10.3390/life12040537 PMC9025914 35455027

[19] Zhang J Liu C Peng Y. Impact of baseline and trajectory of the atherogenic index of plasma on incident diabetic kidney disease and retinopathy in participants with type 2 diabetes: a longitudinal cohort study. Lipids Health Dis. 2024;23:11. 10.1186/s12944-024-02003-5 PMC10782533 38212770

[20] Pivetta B Chen L Nagappa M. Use and performance of the STOP-bang questionnaire for obstructive sleep apnea screening across geographic regions: a systematic review and meta-analysis. JAMA Network Open. 2021;4:e211009. 10.1001/jamanetworkopen.2021.1009 PMC7941199 33683333

[21] Chung F Abdullah HR Liao P. STOP-bang questionnaire: a practical approach to screen for obstructive sleep apnea. Chest. 2016;149:631–8. 10.1378/chest.15-0903 26378880

[22] Qu L Fang S Lan Z. Association between atherogenic index of plasma and new-onset stroke in individuals with different glucose metabolism status: insights from CHARLS. Cardiovasc Diabetol. 2024;23:215. 10.1186/s12933-024-02314-y PMC11193183 38907337

[23] Zheng M Duan X Zhou H. (2023). Association between glycolipids and risk of obstructive sleep apnea: a population-based study. Front Nutr. 2024;9748:01. 10.3389/fnut.2023.974801 PMC10060897 37006942

[24] Li C Peng Y Zhu X. Independent relationship between sleep apnea-specific hypoxic burden and glucolipid metabolism disorder: a cross-sectional study. Respir Res. 2024;25:214. 10.1186/s12931-024-02846-7 PMC11102635 38762509

[25] Agholme J Ahtola K Toll E. Clinically available predictors of obstructive sleep apnoea requiring treatment in type 2 diabetes patients in primary care. Sci Rep. 2025;15:8710. 10.1038/s41598-025-93362-1 PMC11906869 40082634

[26] Gu M Huang W Li X. Association of hypertriglyceridemic waist phenotype with obstructive sleep apnea: a cross-sectional study. Nat Sci Sleep. 2021;13:2165–73. 10.2147/NSS.S335288 PMC8694710 34955662

[27] Thornton JD Dudley KA Saeed GJ. Differences in symptoms and severity of obstructive sleep apnea between black and white patients. Annal Am Thor Soc. 2022;19:272–8. 10.1513/AnnalsATS.202012-1483OC PMC8867366 34242152

[28] Dudley KA Patel SR. Disparities and genetic risk factors in obstructive sleep apnea. Sleep Med. 2016;18:96–102. 10.1016/j.sleep.2015.01.015 PMC4602395 26428843

[29] Ernest DK Chandrasekhar A Xie L Almandoz JP Messiah SE. Relationship between sleep apnea symptoms and metabolic syndrome among racially and ethnically diverse adolescents and young adults in the United States. Obesity Sci Pract. 2025;11:e70071. 10.1002/osp4.70071 PMC11930378 40124958

[30] Miglis MG. Autonomic dysfunction in primary sleep disorders. Sleep Med. 2016;19:40–9. 10.1016/j.sleep.2015.10.001 27198946

[31] Meszaros M Kunos L Tarnoki AD Tarnoki DL Lazar Z Bikov A. The role of soluble low-density lipoprotein receptor-related protein-1 in obstructive sleep apnoea. J Clin Med. 2021;10:1494. 10.3390/jcm10071494 PMC8038392 33916750

[32] Assempoor R Daneshvar MS Taghvaei A. Atherogenic index of plasma and coronary artery disease: a systematic review and meta-analysis of observational studies. Cardiovasc Diabetol. 2025;24:35. 10.1186/s12933-025-02582-2 PMC11756160 39844262

[33] Hang F Chen J Wang Z Zheng K Wu Y. Association between the atherogenic index of plasma and major adverse cardiovascular events among non-diabetic hypertensive older adults. Lipids Health Dis. 2022;21:62. 10.1186/s12944-022-01670-6 PMC9308240 35869550

[34] Bikov A Meszaros M Kunos L Negru AG Frent SM Mihaicuta S. Atherogenic index of plasma in obstructive sleep apnoea. J Clin Med. 2021;10:417. 10.3390/jcm10030417 PMC7865393 33499142

[35] Xu S Wan Y Xu M. The association between obstructive sleep apnea and metabolic syndrome: a systematic review and meta-analysis. BMC Pulm Med. 2015;15:105. 10.1186/s12890-015-0102-3 PMC4578823 26391008

[36] Heffernan A Duplancic D Kumric M Ticinovic Kurir T Bozic J. Metabolic crossroads: unveiling the complex interactions between obstructive sleep apnoea and metabolic syndrome. Int J Mol Sci . 2024;25:3243. 10.3390/ijms25063243 PMC10969858 38542217

[37] Rabiee Rad M Ghasempour Dabaghi G Darouei B Amani-Beni R. The association of atherogenic index of plasma with cardiovascular outcomes in patients with coronary artery disease: a systematic review and meta-analysis. Cardiovasc Diabetol. 2024;23:119. 10.1186/s12933-024-02198-y PMC10986012 38566139

[38] Orrù G Storari M Scano A Piras V Taibi R Viscuso D. Obstructive sleep apnea, oxidative stress, inflammation and endothelial dysfunction-an overview of predictive laboratory biomarkers. Eur Rev Med Pharmacol Sci. 2020;24:6939–48. 10.26355/eurrev_202006_21685 32633387

[39] Chen J Lin S Zeng Y. An Update on obstructive sleep apnea for atherosclerosis: mechanism, diagnosis, and treatment. Front Cardiovasc Med. 2021;8:647071. 10.3389/fcvm.2021.647071 PMC8060459 33898538

[40] Malhotra A Huang Y Fogel R. Aging influences on pharyngeal anatomy and physiology: the predisposition to pharyngeal collapse. Am J Med. 2006;119:72.e9–14. 10.1016/j.amjmed.2005.01.077 PMC2287192 16431197

[41] Shaker R Lang IM. Effect of aging on the deglutitive oral, pharyngeal, and esophageal motor function. Dysphagia. 1994;9:221–8. 10.1007/BF00301914 7805420

[42] Gao F Li YR Xu W. Upper airway morphological changes in obstructive sleep apnoea: effect of age on pharyngeal anatomy. J Laryngol Otol. 2020;134:354–61. 10.1017/S0022215120000766 32284084

[43] Eikermann M Jordan AS Chamberlin NL. The influence of aging on pharyngeal collapsibility during sleep. Chest. 2007;131:1702–9. 10.1378/chest.06-2653 PMC2278166 17413053

[44] Hudgel DW Devadatta P Hamilton H. Pattern of breathing and upper airway mechanics during wakefulness and sleep in healthy elderly humans. J Appl Physiol (Bethesda, Md. : 1985). 1993;74:2198–204. 10.1152/jappl.1993.74.5.2198 8335548

[45] Edwards BA O’Driscoll DM Ali A Jordan AS Trinder J Malhotra A. Aging and sleep: physiology and pathophysiology. Semin Respir Crit Care Med. 2010;31:618–33. 10.1055/s-0030-1265902 PMC3500384 20941662

[46] Mutlu AS Duffy J Wang MC. Lipid metabolism and lipid signals in aging and longevity. Dev Cell. 2021;56:1394–407. 10.1016/j.devcel.2021.03.034 PMC8173711 33891896

[47] Alvarez-Jimenez L Morales-Palomo F Moreno-Cabañas A Ortega JF Mora-Rodríguez R. Effects of statin therapy on glycemic control and insulin resistance: a systematic review and meta-analysis. Eur J Pharmacol. 2023;947:175672. 10.1016/j.ejphar.2023.175672 36965747

[48] Lee SH Kim HS Park YM. HDL-cholesterol, its variability, and the risk of diabetes: a nationwide population-based study. J Clin Endocrinol Metab. 2019;104:5633–41. 10.1210/jc.2019-01080 31408161

[49] Gu Y Li Z Han X. Smoking, alcohol consumption, and risk of arterial stiffness: a two-sample mendelian randomization study. Rev Cardiovasc Med. 2024;25:255. 10.31083/j.rcm2507255 PMC11317346 39139409

[50] Landeo-Gutierrez J Ryu J Tantisira K Bhattacharjee R. Ethnic/racial and sex disparities in obstructive sleep apnea among adolescents in southern California. J Clin Sleep Med. 2024;20:1637–45. 10.5664/jcsm.11238 PMC11446114 38913342

